# Relationship between Postoperative Pain and Sociocultural Level in Major Orthopedic Surgery

**DOI:** 10.1155/2022/7867719

**Published:** 2022-10-11

**Authors:** Bárbara Gouveia, Sara Fonseca, Daniel Humberto Pozza, Daniela Xará, André Sá Rodrigues

**Affiliations:** ^1^Department of Anesthesiology, University Hospital São João, Porto, Portugal; ^2^Department of Biomedicine, Faculty of Medicine and Department of Histology, Faculty of Nutrition and Food Sciences, and Institute for Research and Innovation in Health-I3s, University of Porto, Porto, Portugal; ^3^Department of Orthopedics, Hospital of Prelada, Porto, Portugal

## Abstract

**Background:**

Total knee arthroplasty (TKA) and total hip arthroplasty (THA) are associated with moderate to severe postoperative pain (POP). POP is theoretically predictable and may be influenced by sociocultural differences. This study aimed to identify the relationship between POP and the sociocultural level of the patient undergoing THA or TKA.

**Methods:**

Prospective study, involving informed-consenting adults conducted through consulting patient's clinical processes, preoperative and postoperative questionnaires. Demographic and anthropometric data, type of surgery, *ASA* classification, sociocultural level of the patient, and POP were assessed.

**Results:**

95 patients, all Caucasian and natural from the north of the Portugal, were included. Younger women undergoing TKA reported higher levels of POP. In females, the ASA 3 physical condition was also associated with higher mean pain intensity. Patients with preoperative chronic pain, without depression diagnosis, and unsatisfied with the current profession showed higher levels of reported POP. Retirees, with lower school degree, reported higher levels of minimal pain.

**Conclusions:**

Job satisfaction, type of surgery, body mass index, presence of chronic pain, and the absence of depression were identified as the main predictors of pain after THA or TKA.

## 1. Introduction

Pain is defined as a multidimensional experience (biophysiological, biochemical, psychosocial, behavioural, and moral variants) associated with, or resembling that associated with, actual or potential tissue damage [1]. Following the American Pain Society recommendations, pain was considered by the Portuguese General Directorate of Health as the 5th vital sign in 2003, being recognized as a good clinical practice to all health care services [2].

According to the International Association for Study of Pain (IASP), acute pain is a pain of recent onset and likely limited duration, usually with a temporal and/or causal definition, being the result of nociceptive system activation and having a protective function (alert and defense). Postoperative pain (POP) is one of the most frequent types of acute pain, being a model of study in this area [1, 3, 4].

Pain is still one of the symptoms most frequently reported by patients in the postoperative period. Unmanaged POP can significantly interfere with morbidity and mortality, hospital discharge, quality of life, and daily activities [5, 6]. The transition from peripheral to central sensitization is one of the most worrying consequences associated with the presence of severe acute pain. Central sensitization changes brain plasticity, which may facilitate the transmission of noxious stimuli, limiting the efficacy of pharmacological agents and increasing the risk of chronic pain development [4, 6, 7]. In this context, identification of acute pain predictive factors allows an earlier intervention [5–7], with consequent reduction of short- and long-term morbidity, use of medication, sick leave, or residual disability [8, 9].

Several authors have found an association between sociodemographic, clinical, and psychological factors, namely, preoperative anxiety and POP intensity [7, 8, 10]. In fact, pain comprises emotional, cognitive, and sensorial components, being the self-description by the patient who feels it, the main indicator of its experience. Therefore, despite being a complex experience influenced by multiple variables, the perception, intensity, and responses associated with pain are necessarily influenced by the sociocultural context [11–13]. Furthermore, pain is defined as a physiological and psychological experience, culturally defined, and each culture has its own language associated with the painful experience [11].

Major orthopedic surgery, including total knee arthroplasty (TKA) and total hip arthroplasty (THA), if successfully performed, relieves functional limitations and pain in advanced stages of osteoarthritis. However, this procedure is associated with moderate to severe POP, as the result of intense nociceptive stimulation [8, 14–16]. In addition, patients with particular genetic diseases, such as autosomal recessive disorder of metabolism, that compromises spine and large joints, can experience particular postoperative pain, even greater than that of healthy people [17]. It was also reported that perception of POP may be different in elderly people who have previously suffered from osteoporotic hip fractures [18]. Maximizing the surgical procedure allows reducing the time of surgery and may reduce postoperative complications such as pain [16]. Although the POP can theoretically be predictable, in practice there is a marked interindividual variation, which may be the result of sociocultural differences [14].

The present study aimed to identify the relationship of POP after major orthopedic surgery (TKA or THA) and the patient´s sociocultural level.

## 2. Methods

This study has been reviewed and received ethical clearance from the Ethics and Research Committee of the São João Hospital under protocol number 250-14, with data collection carried out between September and December of 2014. The study cohort consisted of patients admitted to elective surgeries of THA and TKA in the São João Hospital. Exclusion criteria were defined as follows: age <18 years, inability to give informed consent, inability to understand Portuguese language, refusal to participate, physical status classification ASA > 3, allergy to analgesics, peptic disease, and previous surgery in the same anatomical site. All patients read and signed the written informed consent.

The data collection was obtained by consulting the patient's clinical charts and by applying two validated questionnaires. The preoperative questionnaire was carried out up to 24 hours before surgery, aiming to identify the demographic and anthropometric variables, type of surgery, ASA physical status, variables related to the patient's sociocultural level (ethnicity, geographical distribution, and schooling), profession (employee, unemployed, and retired), satisfaction with the profession, desired profession, and lifestyles (sports practice and frequency of sports practice). The presence of clinical diagnosis of depression/depressive syndrome, pharmacological treatment for depression/depressive syndrome, depressive profile, and chronic pain were also evaluated. The postoperative questionnaire was obtained 48 hours after surgery and aimed to evaluate the dependent variables and POP intensity. For this purpose, the Numerical Scale (NS) was used (0 no pain, 1 to 3 mild pain, 4 to 6 moderate pain, 7 to 9 severe pain, and 10 worst pain imaginable). The evaluation of minimal, average, and maximum pain, as well as the current pain, according to the Brief Pain Inventory (BPI) was also performed. Data were collected by the attending anesthetists previously trained in order to improve data quality and avoid bias during the patient interview. Statistical analysis was performed using SPSS® software version 22.0 (IBM Corporation, New York, USA).

Numerical variables were summarized by mean ± standard deviation (SD) and qualitative variables were synthesized using absolute and relative frequencies.

### 2.1. Statistical Analysis

The nonparametric Mann–Whitney test (in the variables with two categories) and Kruskal–Wallis nonparametric test (in the variables with three or more categories) were used in the analysis of the minimum, average, maximum, and current pain levels. A multiple linear regression was performed to determine the significant predictors of the minimum, average, maximum, and current POP. The predictivity used in these regressions was those that in the univariate analysis presented a *p* value <0.200. All calculated *p* values referred to bilateral probabilities, and *p* values <0.05 were considered statistically significant.

## 3. Results

The results comprised 95 patients, all Caucasian and Northerners, 58 underwent TKA and 37 underwent THA. Descriptive data characterizing the sample are described in Table 1.

Women reported higher average (4.84 ± 1.59) and maximum pain levels (6.97 ± 2.19). Patients who underwent TKA presented higher average (5.05 ± 1.42) and maximum (7.12 ± (2)) pain levels (Table 2). There were no statistical differences in the pain intensity regarding age, body mass index (BMI), and *ASA* physical status.

The sociocultural variables (ethnicity, geographic distribution, schooling, current profession, and lifestyles) did not influence the intensity of the POP. However, retirees with only basic education reported higher levels of minimum pain (2.15 ± 1.87) (Table 2). In the evaluation of satisfaction related to the profession, it can be observed that patients not satisfied with the profession had higher medium (6 ± 0) and maximum (8 ± 0) pain scores (Table 2).

Patients with clinical diagnosis of depression reported higher maximum pain (7.47 ± 2.27) and lower pain at the time of the interview (1.94 ± 2.5). Patients with chronic pain history reported higher average pain (4.89 ± 1.63) and also higher pain at the time of the interview (3.09 ± 2.66) (Table 2). There were no changes in the POP intensity regarding the depressive profile, medication for depression, or in relation to the desired profession by the patients (*p* > 0.05).

When the pain intensity was related to the type of surgery, considering gender and age, it was demonstrated that women and young patients (regardless of sex) who underwent TKA showed higher average (5.17 ± 1.39 and 4.9 ± 1.27) and maximum pain scores (7.36 ± 2.00 and 6.94 ± 1.88) than women and young people who underwent THA (Table 3).

Women having depression and physical status ASA 3 reported higher maximum pain intensity (7.47 ± 2.27 and 8.88 ± 1.25, respectively). Physical status ASA 3 in women was also associated with a higher average pain intensity (6.13 ± 1.25) (Table 3).

In the multiple linear regression analysis, patients totally satisfied and patients submitted to THA had lower levels of minimum pain than those satisfied (*p*=0.025), very satisfied (*p*=0.016), and also than those patients underwent TKA (*p*=0.018). Totally satisfied patients had lower levels of maximum pain intensity than very satisfied patients (*p*=0.002). BMI was the only variable that significantly predicted the average POP, and patients with BMI ≥35 presented higher levels of POP (Table 4).

When the pain in the moment of the interview was evaluated, there were two variables that significantly predicted it: depression and chronic pain. Patients without depression (*p*=0.006) and with chronic pain reported higher levels of pain (*p*=0.008) (Table 4).

## 4. Discussion

The main results of this prospective study demonstrated that POP may be influenced by age, gender, type of surgery, history of chronic pain, psychological, and sociocultural aspects. Differences in pain perception seem to be related to cultural, educational, and genetic diseases, but the literature remains controversial [17, 19, 20]. Since POP is subjective and multifactorial, any analysis should be performed considering these limitations.

Despite allowing the relieve functional limitations and pain in advanced stages of osteoarthritis, major orthopedic surgery can be associated with moderate to severe POP [8, 14–16]. Among the predictive factors of POP after orthopedic surgery reported in the literature, the most consistently identified are the patient's age, genetic condition, type of surgery, modality of surgery, previous chronic pain, and preoperative anxiety [5, 6, 14, 16–18, 21–23]. Additionally, psychological factors, namely, optimism and preoperative mental health as well as preoperative pain were identified as predictors of POP after TKA and THA [7, 21, 23]. These factors should be considered in the individualization of analgesia for the high-risk patients.

In a systematic review comprising 23037 patients undergoing various types of surgeries (gastrointestinal, gynecologic, and orthopedic surgeries), age was reported as one of the main factors that influenced POP. However, gender was not related to differences in POP [5]. In a cohort study, age and gender only influenced the functional outcome after TKA, and there was no relationship between these variables and the intensity of POP [23]. On the other hand, differences in gender could be related to psychosocial factors and biological mechanisms that may justify greater sensitivity to the nociceptive stimulus and lower level of endogenous stress-induced analgesia in women [19]. In the present study, gender influenced the mean and maximum POP, since women reported higher pain levels. Women who underwent TKA, with physical status ASA 3, had more severe average and maximum pain and those with diagnosis of depression reported only higher intensity of maximum pain. It is important to consider that this fact can be culturally influenced. Nevertheless, these findings are in accordance with previous studies in Italy [24], the Netherlands [25], and Honk Kong, where females also reported higher levels of current pain and worst pain intensities [26].

Previous studies suggested a lower frequency and intensity of pain in elderly individuals [25, 27]. In the present study, age was not predictive of POP intensity. Although the mean age of the studied patients was lower than 65 years, older patients did not report less pain. More specifically, the previous studies used different age cutoffs and found that significantly higher proportions of younger patients (<60 years) reported moderate or severe pain [25]; and younger patients (<65 years) undergoing TKA reported higher average and maximum pain levels [27].

Orthopedic surgery, involving the large joints, spine, thoracic, and abdominal surgeries, is among the procedures usually associated with higher levels of pain intensity. Furthermore, POP is often inadequately managed in clinical practice [2, 4–6, 8, 22]. This is an important aspect in decision-making concerning analgesic treatment [5, 6, 25, 26]. In accordance with the results of the present study, a previous comparative study of POP in THA and TKA was conducted in 92 patients in Portugal. TKA was also associated with more severe and persistent POP and slower recovery time [22]. The authors also found an association between TKA and higher intensity of average and maximum pain in the studied population. Along with a better analgesic regimen, maximizing the surgical procedure is likely to reduce postoperative complications such as pain [16].

ASA physical status is one of the factors that should be considered when planning the surgery. The present study evaluates only patients with ASA 3 or less, and when assessed separately, ASA classification did not interfere with pain intensity. However, the ASA 3 physical status in women was associated with more severe average and maximum pain. A previous study found ASA as a predictor of POP, with worse physical states (ASA >= 3) associated with more severe pain on the first day after surgery [28].

The body mass index is also another variable that can influence the pain in the joints and POP, being a challenging task in analgesic management, mainly for those with a high BMI [29]. In the present study, BMI was one of the predictors of POP after THA or TKA. On the other hand, some previous studies failed to prove BMI as a major preoperative predictor of pain after TKA [23]. Therefore, pain should be evaluated under its multidimensional experience [1].

The socioeconomic level (defined by the Index of Relative Advantage and Disadvantage that incorporates variables such as salary, education, employability, and occupation) was not previously found as an independent predictor of pain and functional outcomes after large joint arthroplasties [21]. In the present study, the retirees with lower educational levels reported more severe pain in variable minimum POP. Individuals fully satisfied with their profession reported lower average and maximum POP levels, and professional satisfaction was identified as a predictive factor for POP. These are aspects that should be considered in the analgesic management, mainly for those patients at high risk of developing chronic postoperative pain.

Psychological factors, namely, preoperative anxiety and optimism have been reported as having a significant influence in the intensity of pain and anxiety after THA and TKA [7]. Furthermore, patients with preoperative optimism usually have more tolerance and lower pain sensitivity [7, 30]. Additionally, anxiety and depression were also associated as predictive factors of POP in patients undergoing TKA [23]. In the present study, depression was identified as a negative predictive factor of POP. Interestingly, depression was associated not only to higher maximum pain intensity but also to lower current pain. On the other hand, playing sports, working in the desired profession, and being medicated for depression were variables not associated with POP intensity.

The chronic inflammation because of joint degeneration leads to chronic pain [16, 31]. Several studies have reported the existence of preoperative pain and/or chronic pain as predictors of more severe POP [5, 6, 13]. Our study also found an association between the presence of chronic pain in the preoperative period and the development of more severe average and current pain. Chronic pain affects the nervous system, facilitating pain [4, 7] and complicating the postoperative analgesia, being identified as an important predictor of POP.

Among the limitations, the results of the present study should be interpreted with caution, as it included a convenience sample size and all patients are Caucasian and treated in the same hospital. The low educational level of the patients may also affect external validity. Generalization of the findings to the population must be done with care.

## 5. Conclusion

POP is multifactorial and influenced by many variables, as demonstrated in the present and in previous studies. Patients with chronic preoperative pain, without a diagnosis of depression, with severe or morbid obesity, undergoing TKA, and who are not completely satisfied with the profession may benefit from individualized analgesic regimens for better pain relief in the first 48 hours after surgery, improving functional recovery.

## Figures and Tables

**Table 1:** Characterization of the study sample (*n* = 95).

Variable		Number	Percentage
Sex	Female	68	71.6
	Male	27	28.4

Age	<65 years	54	56.8
	≥65 years	41	43.2

Schooling	No studies	9	9.5
	1^st^ cycle of basic education	68	71.6
	2^nd^ cycle of basic education	5	5.3
	3^rd^ cycle of basic education	7	7.4
	Secondary school	1	1.1
	Graduation	5	5.3

Profession	Unemployed	3	3.2
	Employed	48	50.5
	Retired	44	46.3

Satisfaction with the profession	None	1	1.1
	Unsatisfied	7	7.4
	Satisfied	19	20.0
	Very satisfied	51	53.7
	Totally satisfied	17	17.9

Desired profession	Desired	66	69.5
	Another one	29	30.5

BMI	≤18	2	2.1
	>18 *e* < 25	15	15.8
	≥25 *e* < 30	43	45.3
	≥30 *e* < 35	27	28.4
	≥35	8	8.4

Sports practice	No	88	92.6
	Yes	7	7.4

Frequency of sports practice	None	88	92.6
	≥=1 times/week	7	7.6

Diagnosis of depression	No	59	62.1
	Yes	36	37.9

Pharmacological treatment of depression	No	6	16.7
	Yes	30	83.3

Depressive profile	No	57	96.6
	Yes	2	3.4

ASA physical status	1	3	3.3
	2	75	81.5
	3	13	14.1

Type of surgery	TKA	58	61.1
	THA	37	38.9

Chronic pain	No	25	26.3
	Yes	70	73.7

**Table 2:** Comparison of postoperative pain according to patient variables (*n* = 95).

Variable		Minimum POP	*p -*Value	Mean POP	*p -*Value	Maximum POP	*p -*Value	Current POP	*p -*Value
Sex	Female	1.47 ± 1.77 (0-6)	0.717	4.84 ± 1.59 (0-8)	**0.020** ^*∗*^	6.97 ± 2.19 (3-10)	**0.010** ^*∗*^	2.49 ± 2.71 (0-9)	0.238
	Male	1.15 ± 1.26 (0-4)		4.04 ± 1.81 (1-8)		5.74 ± 1.87 (3-10)		3.19 ± 2.59 (0-7)	

Type of surgery	THA	0.89 ± 1.17 (0-4)	0.052	3.92 ± 1.85 (0-8)	**0.002** ^*∗*^	5.84 ± 2.20 (3-10)	**0.005** ^*∗*^	2.84 ± 2.76 (0-8)	0.725
	TKA	1.69 ± 1.83 (0-6)		5.05 ± 1.42 (2-8)		7.12 ± 2.00 (3-10)		2.58 ± 2.65 (0-9)	

Current work	Unemployed	0.00 ± 0.00	**0.004** ^*∗*^	3.50 ± 0.71	0.114	4.50 ± 2.12	0.410	4.00 ± 2.83	0.425
	Employed	0.85 ± 1.37		4.03 ± 1.53		6.55 ± 2.14		2.97 ± 2.91	
	Retired	2.15 ± 1.87		4.85 ± 1.79		6.12 ± 1.87		2.27 ± 2.60	

Satisfaction with the profession	Unsatisfied	2.00 ± 0.00 (2-2)	0.178	6.00 ± 0.00 (6-6)	**0.002** ^*∗*^	8.00 ± 0.00 (8-8)	**0.003** ^*∗*^	0.00 ± 0.00 (0-0)	0.067
	Satisfied	1.95 ± 1.75 (0-5)		5.26 ± 1.73 (1-8)		6.58 ± 1.92 (3-10)		2.89 ± 2.35 (0-7)	
	Very satisfied	1.47 ± 1.78 (0-6)		4.86 ± 1.59 (0-8)		7.27 ± 2.02 (3-10)		3.06 ± 2.85 (0-9)	
	Totally satisfied	0.76 ± 1.09 (0-3)		3.41 ± 1.28 (2-5)		5.06 ± 2.11 (3-10)		2.44 ± 2.63 (0-7)	

Diagnosis of depression	No	1.41 ± 1.6 (0-6)	0.655	4.37 ± 1.81 (0-8)	0.070	6.10 ± 1.94 (3-10)	**0.003** ^*∗*^	3.14 ± 2.71 (0-8)	**0,048** ^*∗*^
	Yes	1.33 ± 1.74 (0–6)		5.00 ± 1.39 (2-8)		7.47 ± 2.27 (3-10)		1.94 ± 2.50 (0-9)	

Chronic pain	No	0.92 ± 1.50 (0-6)	0.078	3.84 ± 1.62 (2-8)	**0.003** ^*∗*^	5.92 ± 2.31 (3-10)	0.051	1.50 ± 2.43 (0-7)	**0,007** ^*∗*^
	Yes	1.54 ± 1.67 (0-6)		4.89 ± 1.63 (0-8)		6.87 ± 2.07 (3-10)		3.09 ± 2.66 (0-9)	

POP: postoperative pain, TKA: total knee arthroplasty, THA: total hip arthroplasty, ^*∗*^*p* < 0,05.

**Table 3:** Comparison of postoperative pain in females and younger patients (*n* = 95).

Variable		Minimum POP	*p -*Value	Mean POP	*p -*Value	Maximum POP	*p -*Value	Current POP	*p -*Value
Female	THA	7.00 ± 0.00	0.159	4.10 ± 1.79	**0.025** ^*∗*^	6.10 ± 2.36	**0.033** ^*∗*^	2.67 ± 2.90	0.777
	TKA	1.72 ± 1.93		5.17 ± 1.39		7.36 ± 2.00		2.40 ± 2.64	

<65 years	THA	0.87 ± 1.14	0.258	3.87 ± 1.66	**0.029** ^*∗*^	5.78 ± 2.02	**0.032** ^*∗*^	2.83 ± 2.89	0.583
	TKA	1.52 ± 1.81		4.9 ± 1.27		6.94 ± 1.88		2.30 ± 2.81	

Female	No depression	1.63 ± 1.83	0.489	4.66 ± 1.79	0.461	6.41 ± 1.97	**0.035** ^*∗*^	3.09 ± 2.84	0.125
	Depression	1.33 ± 1.74		5.00 ± 1.39		7.47 ± 2.27		1.94 ± 2.50	

Female	ASA 1	0.00 ± 0.00	0.455	2.00 ± 0.00	**0.015** ^*∗*^	3.00 ± 0.00	**0.010** ^*∗*^	6.00 ± 0.00	0.262
	ASA 2	1.43 ± 1.70		4.74 ± 1.53		6.83 ± 2.11		2.55 ± 2.73	
	ASA 3	2.13 ± 2.36		6.13 ± 1.25		8.88 ± 1.25		1.75 ± 2.55	

POP: postoperative pain, TKA: total knee arthroplasty, THA: total hip arthroplasty, ^*∗*^*p* < 0,05.

**Table 4:** Multiple linear regression analysis of postoperative pain (*n* = 95).

	Minimum POP		Mean POP		Maximum POP		Current POP	
	B (IC 95%)	*p*	B (IC 95%)	*p*	B (IC 95%)	*p*	B (IC 95%)	*p*
Constant	4.94 (3.29/6.59)	0.001	3.19 (1.38/5.01)	0.001	7.71 (2.60/12.82)	0.004	1.80 (0.25/3.36)	0.024
Satisfied with profession	**1.24 (0.16/2.32)**	**0.025** ^*∗*^	0.99 (−0.11/2.09)	0.077	1.32 (−0.08/2.72)	0.064	0.30 (−1.43/2.03)	0.732
Very satisfied with profession	**1.10 (0.22/1.99)**	**0.016** ^*∗*^	0.48 (−0.42/1.38)	0.292	**1.90 (0.74/3.06)**	**0.002** ^*∗*^	0.79 (−0.66/2.25)	0.282
BMI < 18			−**2.64 (−5.24//0.04)**	**0.047** ^*∗*^				
BMI > 18 *e* < 25			−**2.22 (**−**3.66//0.77)**	**0.003** ^*∗*^				
BMI ≥ 25 *e* < 30			−**1.56 (**−**2.86//0.25)**	**0.020** ^*∗*^				
BMI ≥30 *e* < 35			−1.13 (−2.49/0.23)	0.103				
Depression	0.08 (−0.69/0.84)	0.847			−0.71 (−1.69/0.28)	0.156	−**1.57 (0.46/2.69)**	**0.006** ^*∗*^
Type of surgery THA	−**0.84 (**−**1.53//0.15)**	**0.018** ^*∗*^			−0.67 (−1.59/0.26)	0.156		
Chronic pain	0.55 (−0.21/1.32)	0.155	0.31 (−0.47/1.09)	0.429	0.10 (−0.90/1.10)	0.848	**1.71 (**−**2.96//0.47)**	**0.008** ^*∗*^
*R * ^2^	0.280		0.210		0.385		0.201	

POP: postoperative pain, THA: total hip arthroplasty, ^*∗*^*p* < 0,05.

## Data Availability

The data used to support the findings of this study are included within the article.

## References

[1] Raja S. N., Carr D. B., Cohen M. (2020). The revised International Association for the Study of Pain definition of pain: concepts, challenges, and compromises. *Pain*.

[2] Pozza D. H., Azevedo L. F., Castro Lopes J. M. (2021). Pain as the fifth vital sign-A comparison between public and private healthcare systems. *PLoS One*.

[3] Apfelbaum J. L., Chen C., Mehta S. S., Gan T. J. (2003). Postoperative pain experience: results from a national survey suggest postoperative pain continues to be undermanaged. *Anesthesia & Analgesia*.

[4] Kehlet H., Dahl J. B. (1993). Postoperative pain. *World Journal of Surgery*.

[5] Ip H. V., Abrishami A., Peng P. H., Wong J., Chung F. (2009). Predictors of postoperative pain and analgesic consumption: a qualitative systematic review. *Anesthesiology*.

[6] Kehlet H., Jensen T. S., Woolf C. J. (2006). Persistent postsurgical pain: risk factors and prevention. *The Lancet*.

[7] Pinto P. R., McIntyre T., Ferrero R., Almeida A., Araujo-Soares V. (2013). Predictors of acute postsurgical pain and anxiety following primary total hip and knee arthroplasty. *The Journal of Pain*.

[8] Strassels S. A., Chen C., Carr D. B. (2002). Postoperative analgesia: economics, resource use, and patient satisfaction in an urban teaching hospital. *Anesthesia & Analgesia*.

[9] Werner M. U., Mjobo H. N., Nielsen P. R., Rudin A., Warner D. S. (2010). Prediction of postoperative pain: a systematic review of predictive experimental pain studies. *Anesthesiology*.

[10] Macrae W. A. (2001). Chronic pain after surgery. *British Journal of Anaesthesia*.

[11] Callister L. C. (2003). Cultural influences on pain perceptions and behaviors. *Home Health Care Management & Practice*.

[12] Gatchel R. J. (2004). Comorbidity of chronic pain and mental health disorders: the biopsychosocial perspective. *American Psychologist*.

[13] Azevedo L. F., Costa-Pereira A., Mendonca L., Dias C. C., Castro-Lopes J. M. (2012). Epidemiology of chronic pain: a population-based nationwide study on its prevalence, characteristics and associated disability in Portugal. *The Journal of Pain*.

[14] Robleda G., Sillero-Sillero A., Puig T., Gich I., Banos J. E. (2014). Influence of preoperative emotional state on postoperative pain following orthopedic and trauma surgery. *Revista Latino-Americana de Enfermagem*.

[15] Barrington J. W., Dalury D. F., Emerson R. H., Hawkins R. J., Joshi G. P., Stulberg B. N. (2013). Improving patient outcomes through advanced pain management techniques in total hip and knee arthroplasty. *American Journal of Orthopedics (Belle Mead NJ)*.

[16] Romeo M., Rovere G., Stramazzo L. (2021). Single use instruments for total knee arthroplasty. *Medicinski Glasnik*.

[17] Meschini C., Cauteruccio M., Oliva M. S. (2020). Hip and knee replacement in patients with ochronosis: clinical experience and literature review. *Orthopedic Reviews*.

[18] Stramazzo L., Ratano S., Monachino F., Pavan D., Rovere G., Camarda L. (2021). Cement augmentation for trochanteric fracture in elderly: a systematic review. *Journal of Clinical Orthopaedics and Trauma*.

[19] Wiesenfeld-Hallin Z. (2005). Sex differences in pain perception. *Gender Medicine*.

[20] Chraif M., Fulga D. (2013). Gender differences in pain perception-a pilot study on young Romanian students. *Procedia—Social and Behavioral Sciences*.

[21] Dowsey M. M., Nikpour M., Choong P. F. (2014). Outcomes following large joint arthroplasty: does socio-economic status matter?. *BMC Musculoskeletal Disorders*.

[22] Pinto P. R., McIntyre T., Ferrero R., Araújo-Soares V., Almeida A. (2013). Persistent pain after total knee or hip arthroplasty: differential study of prevalence, nature, and impact. *Journal of Pain Research*.

[23] Judge A., Arden N. K., Cooper C. (2012). Predictors of outcomes of total knee replacement surgery. *Rheumatology*.

[24] Costantini M., Viterbori P., Flego G. (2002). Prevalence of pain in Italian hospitals: results of a regional cross-sectional survey. *Journal of Pain and Symptom Management*.

[25] Sommer M., de Rijke J. M., van Kleef M. (2008). The prevalence of postoperative pain in a sample of 1490 surgical inpatients. *European Journal of Anaesthesiology*.

[26] Chung J. W. Y., Lui J. C. Z. (2003). Postoperative pain management: study of patients’ level of pain and satisfaction with health care providers’ responsiveness to their reports of pain. *Nursing and Health Sciences*.

[27] Gibson S. J., Helme R. D. (2001). Age-related differences in pain perception and report. *Clinics in Geriatric Medicine*.

[28] Kinjo S., Sands L. P., Lim E., Paul S., Leung J. M. (2012). Prediction of postoperative pain using path analysis in older patients. *Journal of Anesthesia*.

[29] van Helmond N., Timmerman H., van Dasselaar N. T. (2017). High Body Mass Index is a potential risk factor for persistent postoperative pain after breast cancer treatment. *Pain Physician*.

[30] Geers A. L., Wellman J. A., Helfer S. G., Fowler S. L., France C. R. (2008). Dispositional optimism and thoughts of well-being determine sensitivity to an experimental pain task. *Annals of Behavioral Medicine*.

[31] Lee Y. C. (2013). Effect and treatment of chronic pain in inflammatory arthritis. *Current Rheumatology Reports*.

